# Isolated prenatal foramen ovale closure or restriction presenting after birth: a distinct, under-recognized clinical condition

**DOI:** 10.3389/fped.2026.1753196

**Published:** 2026-06-05

**Authors:** Tomaž Podnar, Ralf Geiger, Ira Winkler, Elke Griesmaier, Susanne Sprung, Ursula Kiechl-Kohlendorfer

**Affiliations:** 1 Department of Pediatrics III (Paediatric Cardiology, Pulmonology, Allergology and Cystic Fibrosis), Medical University of Innsbruck, Innsbruck, Austria; 2 Department of Pediatrics II (Neonatology), Medical University of Innsbruck, Innsbruck, Austria; 3 Innpath, Tirol Kliniken, Innsbruck, Austria

**Keywords:** foramen ovale restriction, left ventricular failure, neonate, persistent pulmonary hypertension in the neonate, persistent pulmonary hypertension of the newborn

## Abstract

**Background:**

The foramen ovale (FO) plays a central role in fetal circulation. Prenatal FO closure or restriction is well-characterized in fetuses and in neonates with congenital heart defects, but is rarely described as an isolated finding after birth. This study aimed to characterize the clinical presentation, echocardiographic features, management, and outcomes of neonates with isolated prenatal FO closure or restriction.

**Methods:**

We retrospectively analyzed 10 consecutive neonates admitted to our tertiary neonatal intensive care unit between September 2018 and August 2025 with FO closure or restriction diagnosed after birth.

**Results:**

The median gestational age was 39 + 1 weeks (range 36 + 4–41 + 1), and the median birth weight was 3,345 g (range 2,600–4,000 g). All the neonates presented within 9 h after birth, nine of them with central or differential cyanosis, seven with associated respiratory distress, and one with apnea. Echocardiography uniformly revealed persistent pulmonary hypertension of the newborn (PPHN) and significantly impaired left ventricular (LV) function with preserved right ventricular (RV) contractility. The interatrial septum was closed in six neonates and was restrictive in four. A prostaglandin infusion was administered to nine neonates. All the patients received inotropic support and pulmonary arterial hypertension (PAH) therapy. In survivors, normalization of LV function occurred in 1–4 days, followed by transition to a predominant left-to-right ductus arteriosus (DA) shunt in 1–8 days. One neonate died from refractory LV failure and pulmonary hemorrhage; one developed global developmental delay.

**Conclusions:**

Isolated prenatal FO closure or restriction represents a distinct and likely under-recognized condition, causing severe neonatal PPHN associated with significant LV failure. These echocardiographic findings, together with preserved RV function, should prompt careful evaluation of the interatrial septum. Early recognition and hemodynamic support can lead to full recovery in the majority of cases.

## Introduction

The foramen ovale (FO) is central to the normal functioning of fetal circulation. In fetuses, FO closure or restriction may result in congestive cardiac failure, fetal hydrops, or death (1, 2). Prenatal FO closure or restriction in neonates with associated congenital heart defects is a well-recognized risk factor for increased mortality after birth (3, 4). In contrast, isolated prenatal FO closure or restriction diagnosed after birth is rare and its postnatal implications remain poorly defined (5–8). This retrospective analysis aims to define the clinical presentation, echocardiographic features, management, and outcome of this condition in a consecutive cohort.

## Materials and methods

We performed a retrospective, single-center review of all neonates admitted to the neonatal intensive care unit (NICU) of the Medical University of Innsbruck between September 2018 and August 2025. Isolated FO closure or restriction was diagnosed after birth in 10 out of 3,274 admitted neonates (0.3%). During the observation period, 71 neonates with persistent pulmonary hypertension of the newborn (PPHN) (2.1% of admitted neonates) were treated. The inclusion criteria were as follows: (1) presentation with cyanosis and/or respiratory distress within 24 h after birth, (2) exclusion of infection and pulmonary disease, (3) exclusion of congenital heart defect, and (4) echocardiographic evidence of FO closure or restriction. Clinical, echocardiographic, laboratory, and outcome data were extracted from electronic medical records. Ethical approval was obtained from the local ethics committee (approval no. 1195/2025). A precise investigation of the interatrial septum was performed: two-dimensional echocardiography was used to evaluate atrial septal morphology and color flow Doppler echocardiography was used to demonstrate an FO shunt. A minimal, turbulent FO shunt in a newborn with PPHN was defined as restrictive. Furthermore, a meticulous examination of ductus arteriosus (DA) was conducted. Two-dimensional echocardiography and color flow and pulsed wave Doppler echocardiography were used to evaluate both the duct size and the shunt. The severity of pulmonary arterial hypertension was estimated based primarily on the ductus arteriosus (DA) shunt, categorized as predominantly right-to-left, bidirectional, or predominantly left-to-right. PPHN was diagnosed in neonates who had a predominantly right-to-left DA shunt and associated flattening of the interventricular septum. The left ventricular (LV) and right ventricular (RV) contractility was estimated semi-quantitatively using two-dimensional echocardiography and categorized as normal, mildly, moderately, or severely impaired. Mitral regurgitation was graded semi-quantitatively as mild, moderate, or severe using color-flow Doppler echocardiography. Management strategies involved a prostaglandin infusion to maintain DA patency, respiratory and inotropic support, and pulmonary arterial hypertension (PAH) therapy. The main outcome measure was survival. At discharge, neurological outcome was assessed both clinically and with neuroimaging using brain ultrasound (US) and/or brain magnetic resonance imaging (MRI). Any major complications experienced during the admission were summarized.

## Results

The median gestational age was 39 weeks (range 36 + 4–41 + 1) and the median birth weight was 3,345 g (range 2,600–4,000 g) (Table 1). Six neonates were female. All the neonates deteriorated within 9 h after birth, nine presenting with a central or differential cyanosis, seven with associated respiratory distress, and one with apnea. Two neonates required cardiopulmonary resuscitation. The FO was closed in six neonates and restrictive in four. At admission, the DA was wide open (unrestrictive) in all 10 neonates: a predominantly right-to-left shunt was noted in nine neonates and a bidirectional shunt in one neonate. LV contractility was severely impaired in eight neonates and moderately impaired in two. RV contractility was preserved in all the neonates. Mitral regurgitation was severe and moderate in four neonates, respectively, and mild in two. Left-sided heart structures (mitral valve, left ventricle, aortic valve, and aortic isthmus) were not hypoplastic in any patient. The maximal troponin T level ranged from 140 to 1,153 ng/L and the maximal NT-pro BNP level from 8,447 to > 70,000 ng/L. A prostaglandin infusion was administered to nine neonates. In patient 2, the diagnosis was established later, after reappraisal of echocardiograms and she did not receive a prostaglandin infusion. All the neonates received inotropic support, seven received a milrinone infusion, and six were supplemented with additional inotropic therapy. The remaining three neonates did not tolerate a milrinone infusion and were supported with dopamine, dobutamine, and/or adrenaline. PAH was treated in all neonates: oxygen was administered to all of them, which was supplemented with nitric oxide and/or sildenafil (intravenous or oral) in seven neonates, respectively. Intubation and mechanical ventilation were necessary in eight neonates (1–19 days), and two neonates received nasal high-flow therapy. LV function normalized 1 to 4 days after admission and a predominantly left-to-right DA shunt was detected 1 to 8 days after admission. Nine of the 10 neonates survived. Significant complications included intracranial or subdural hemorrhage (*n* = 2), intestinal perforation (*n* = 1), pneumomediastinum (*n* = 1), and lung hemorrhage (*n* = 1). Neurological outcomes were favorable in all but two children, who exhibited developmental delay and focal epilepsy, respectively. A detailed description of patient characteristics, including treatment strategies, is given in Table 1.

**Table 1:** Patient data: presentation, management, and outcome.

Patient data	Patient 1	Patient 2	Patient 3	Patient 4	Patient 5	Patient 6	Patient 7	Patient 8	Patient 9	Patient 10
Gestational age (weeks + days)	39 + 5	39 + 1	40 + 2	41 + 0	40 + 2	37 + 3	41 + 1	38 + 6	36 + 4	39 + 2
Birth weight (g)	3,090	3,500	3,370	4,000	3,345	3,310	3,650	3,210	2,600	3,000
Sex (M/F)	M	F	F	F	M	F	F	M	M	F
Umbilical cord, pH	7.35	7.29	7.37	7.13	7.47	7.30 (venous)	7.28	7.13	7.33	7.47
Umbilical cord, BE (nmol/L)	−2.6	−5.7	−2.3	−5.7	−2.7	−4.3 (venous)	−2.1	−10.3	1.3	−3.1
Apgar score	9/9/9	7/9/10	9/10/10	6/6/7	3/4/4	7/8/7	9/9/10	8/9/10	6/8/9	6/5/4
Onset of deterioration	After 40 min	After 20 min	After 4 h	Instantly	Instantly	Instantly	After 20 min	After 9 h	After 30 min	After 10 min
Clinical presentation	Central cyanosis, respiratory distress	Central cyanosis, respiratory distress	Differential cyanosis	Central cyanosis	Apnea	Central cyanosis, respiratory distress	Central cyanosis, respiratory distress	Differential cyanosis, respiratory distress	Central cyanosis, respiratory distress	Central cyanosis, respiratory distress
Cardio-pulmonary resuscitation	–	–	–	Yes	–	–	–	–	–	Yes
Blood gas analysis at NICU admission, pH	pH 6.98 (venous)	pH 6.77 (arterial)	pH 7.40 (venous)	pH 6.9 (arterial)	pH 7.29 (arterial)	pH 7.11 (venous)	pH 6.79 (arterial)	pH 7.33 (venous)	pH 7.19 (venous)	pH unmeasurable (venous)
Blood gas analysis at NICU admission, lactate (mg/L)	83 (venous)	86 (arterial)	29 (venous)	177 (arterial)	28 (arterial)	45 (venous)	126 (arterial)	35 (venous)	34 (venous)	153 (venous)
Interatrial septum	Closed, thick	Closed, thick	Closed, thick	Restrictive, thin	Restrictive, thin	Closed, thick	Closed, thin	Restrictive, thick	Restrictive, thick	Closed, thick
LV function at NICU admission	Severely impaired	Severely impaired	Severely impaired	Severely impaired	Severely impaired	Moderately impaired	Severely impaired	Moderately impaired	Severely impaired	Severely impaired
Mitral regurgitation at NICU admission	Severe	Severe	Mild	Moderate	Moderate	Severe	Severe	Moderate	Moderate	Moderate
Max. troponin T value (ng/L)	400	1,153	158	886	353	140	797	242	176	1,120
Max. NT-proBNP value (ng/L)	33,019	>70,000	38,445	>70,000	58,564	30,659	50,889	41,292	>70,000	8,447
Inotropic support	Levosimendan, milrinone, dopamine, adrenaline, noradrenaline	Adrenaline	Levosimendan, milrinone	Noradrenaline, dobutamine, milrinone	Milrinone, dopamine	Dopamine	Milrinone, dobutamine	Milrinone	Milrinone, dopamine	Dopamine, dobutamine, adrenaline
Normalization of LV function	After 2 days	After 1 day	After 4 days	After 2 days	After 3 days	After 1 day	After 2 days	After 2 days	After 2 days	–
PAH therapy	Oxygen, NO, bosentan, i.v., p.o., sildenafil i.v., p.o.	Oxygen, NO	Oxygen, sildenafil p.o.	Oxygen, NO, sildenafil i.v. + p.o.	Oxygen, NO, sildenafil i.v., p.o.	Oxygen	Oxygen, NO, sildenafil i.v., p.o.	Oxygen, sildenafil p.o.	Oxygen, NO, sildenafil p.o.	Oxygen, NO
Predominant L–R DA shunt	After 8 days	After 3 days	After 5 days	After 5 days	After 6 days	After 1 day	After 5 days	After 1 day	After 2 days	–
Intubation	12 days	10 days	High-flow	19 days	12 days	1 day	11 days	High-flow	9 days	Until death
Brain MRI/US	MRI normal	US and MRI: intracranial hemorrhage	US normal	MRI: hypoxic-ischemic encephalopathy	MRI normal	US normal	MRI normal	US normal	US normal	–
Complications	–	Intestinal perforation	–	Subdural and lung hemorrhage	Pneumo-mediastinum	–	–	–	–	Lung hemorrhage

BE, base excess; DA, ductus arteriosus; F, female; i.v., intravenous; LV, left ventricular; M, male; MRI, magnetic resonance imaging; NICU, intensive care unit, NO, nitric oxide; PAH, pulmonary arterial hypertension; pH, potential of hydrogen; p.o., orally; US, ultrasound.

## Discussion

The FO is crucial for the normal functioning of fetal circulation and, together with the DA, allows an almost complete bypass of the fetal pulmonary circulation. The crescent-shaped FO opening directs oxygenated blood coming from the placenta into the LV, providing approximately 75% of the LV output. Prenatal FO closure or restriction leads to increased RV output and pulmonary and ductal blood flow, while LV output is correspondingly reduced. Consequently, typical fetal echocardiographic findings include RV dilatation, tricuspid regurgitation, and signs of increased systemic venous pressure (1, 2). In severe cases, markedly increased systemic venous pressure can lead to pericardial effusion, ascites, and/or hydrothorax, and, if progressive, may cause fetal hydrops or fetal death. Initially, a hypermobile or redundant septum primum is almost always observed, presumably contributing to FO restriction or closure (1). However, the cause(s) of prenatal FO closure or restriction are not known.

In 6 of our 10 patients, organ screening was performed between the 20th and 24th weeks of gestation and FO closure or restriction was not detected in any of them. After birth, the left side of the heart (mitral valve, left ventricle, aortic valve, and aorta with aortic isthmus) was not hypoplastic in any of our patients. Therefore, we presume that FO closure or restriction developed near term, causing functional disruption and not left heart hypoplasia.

The reported experience in neonates with prenatal FO closure or restriction is rare and controversial (5). Gupta et al. reported favorable outcomes in a group of seven consecutive neonates with restrictive FO (6). At the initial presentation, none of the neonates was symptomatic and only one patient required PAH treatment with nitric oxide (NO) and sildenafil. Recently, Kadish et al. reported 34 patients with premature FO closure identified *in utero* (7). After birth, FO restriction was demonstrated in 32% of the neonates, while complete FO closure was not confirmed in any. The clinical course in this group of neonates was relatively benign; the most common interventions received were continuous positive airway pressure (CPAP) and intravenous fluids. Only two of these patients required a milrinone infusion and none required inhaled nitric oxide (NO).

In contrast, our experience is consistent with two fetal studies showing that isolated prenatal FO closure or restriction may be associated with a severe clinical course after birth (1, 2, 8). One of our 10 neonates died and cardiopulmonary resuscitation was necessary in one additional neonate. All our patients required NICU treatment, eight of them with intubation and mechanical ventilation. One of our patients was extubated after 1 day, while the remaining patients needed prolonged mechanical ventilation, up to 19 days.

**Figure 1:**
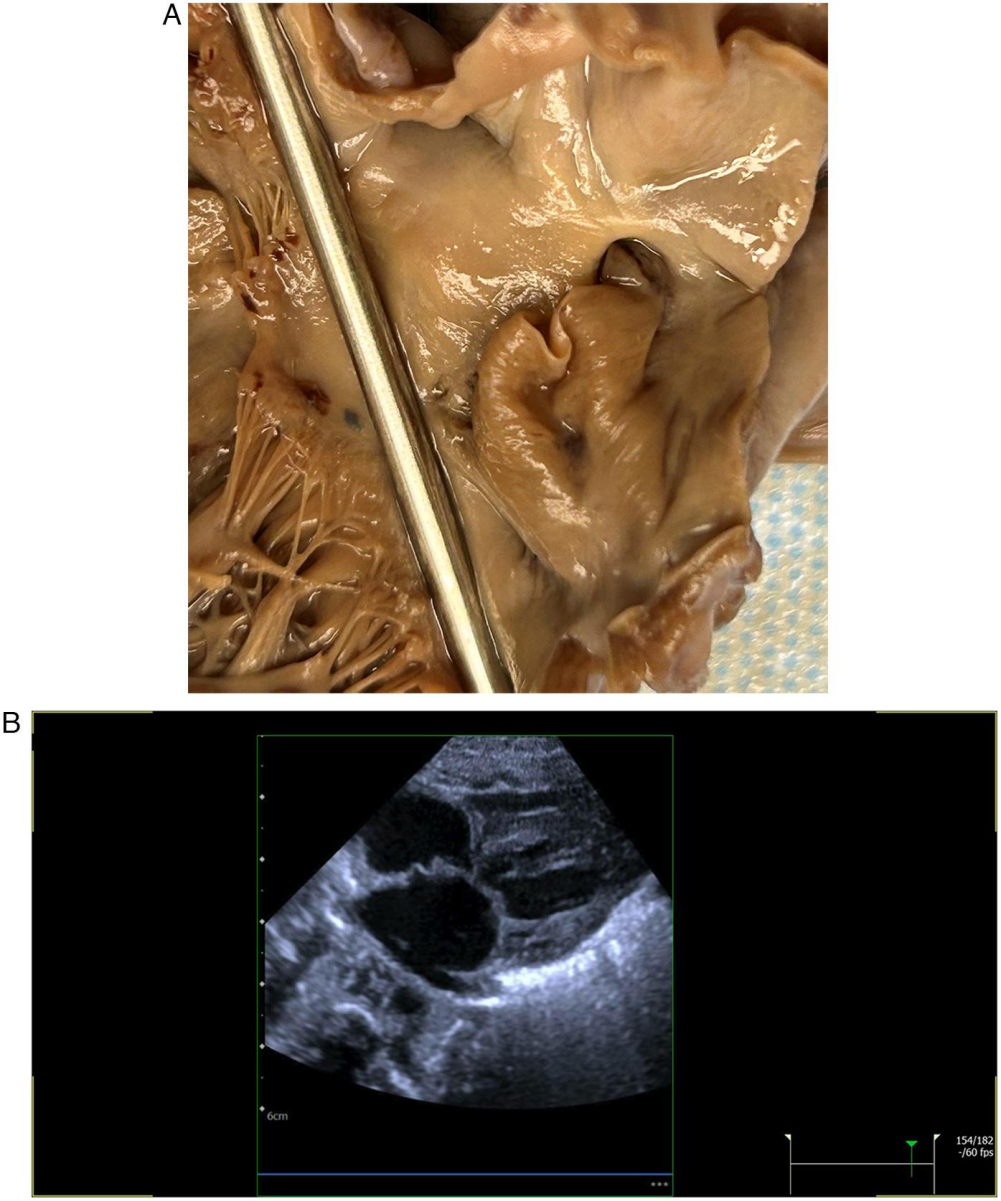
**(A)** Autopsy photograph, left side of the interatrial septum of a thick, aneurysmal interatrial septum, with a small, ectopic opening. **(B)** Transthoracic echocardiography, subcostal four-chamber view, of the thick interatrial septum depicted in **(A)**.

**Figure 2:**
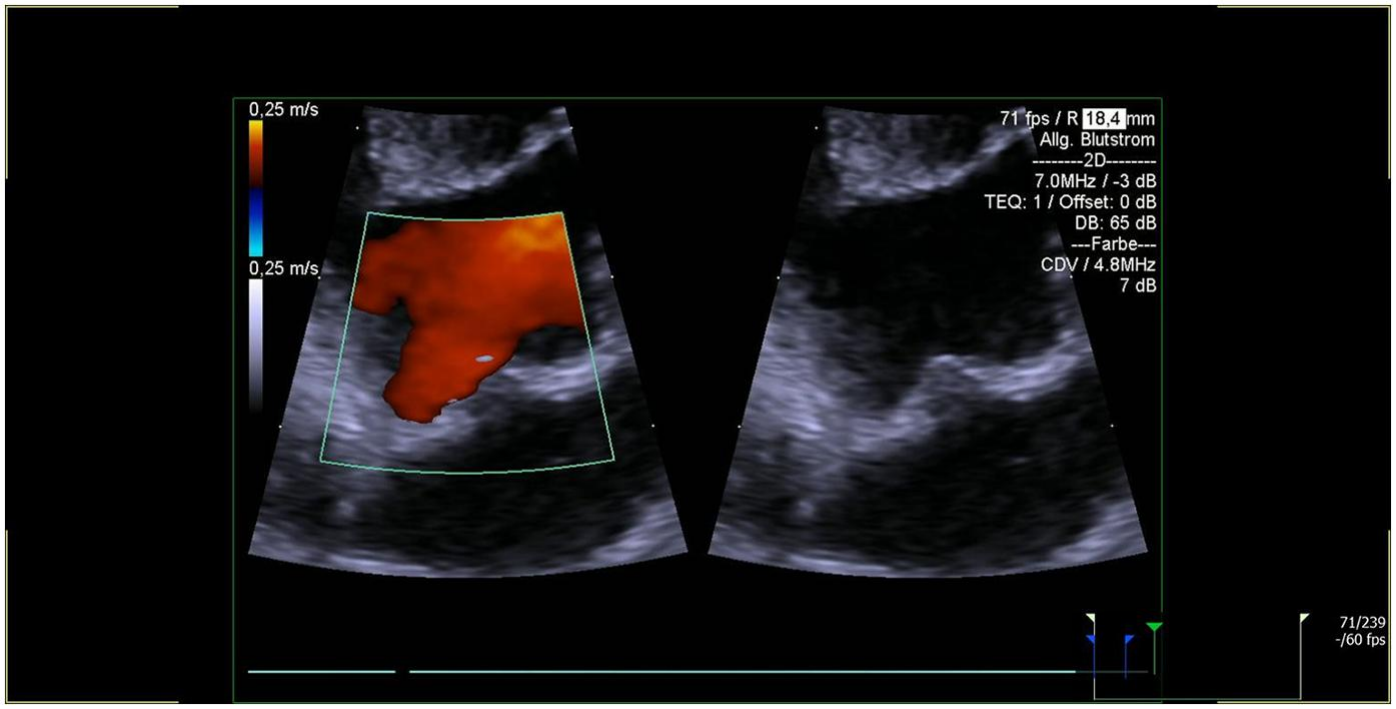
Transthoracic echocardiography, subcostal four-chamber view, of a thick interatrial septum with complete FO closure.

**Figure 3:**
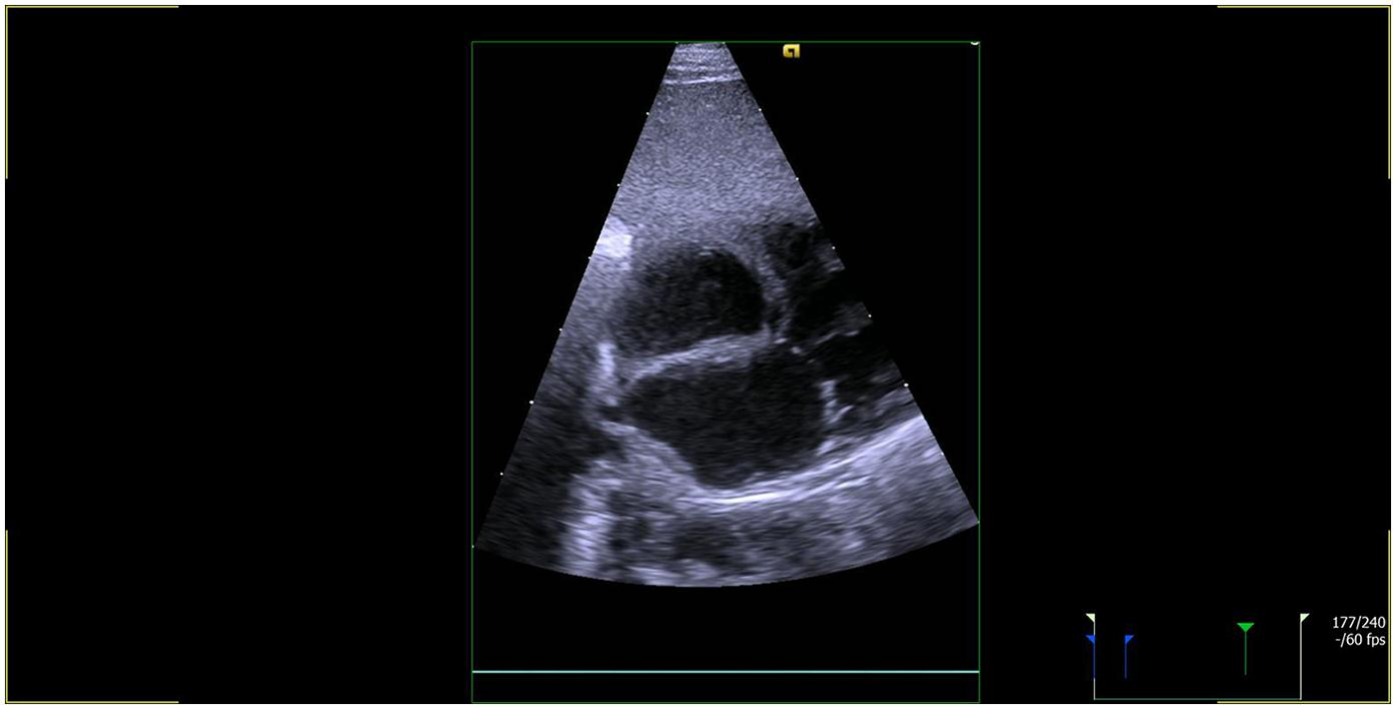
Transthoracic echocardiography, subcostal four-chamber view, of a thick interatrial septum with complete FO closure.

**Figure 4:**
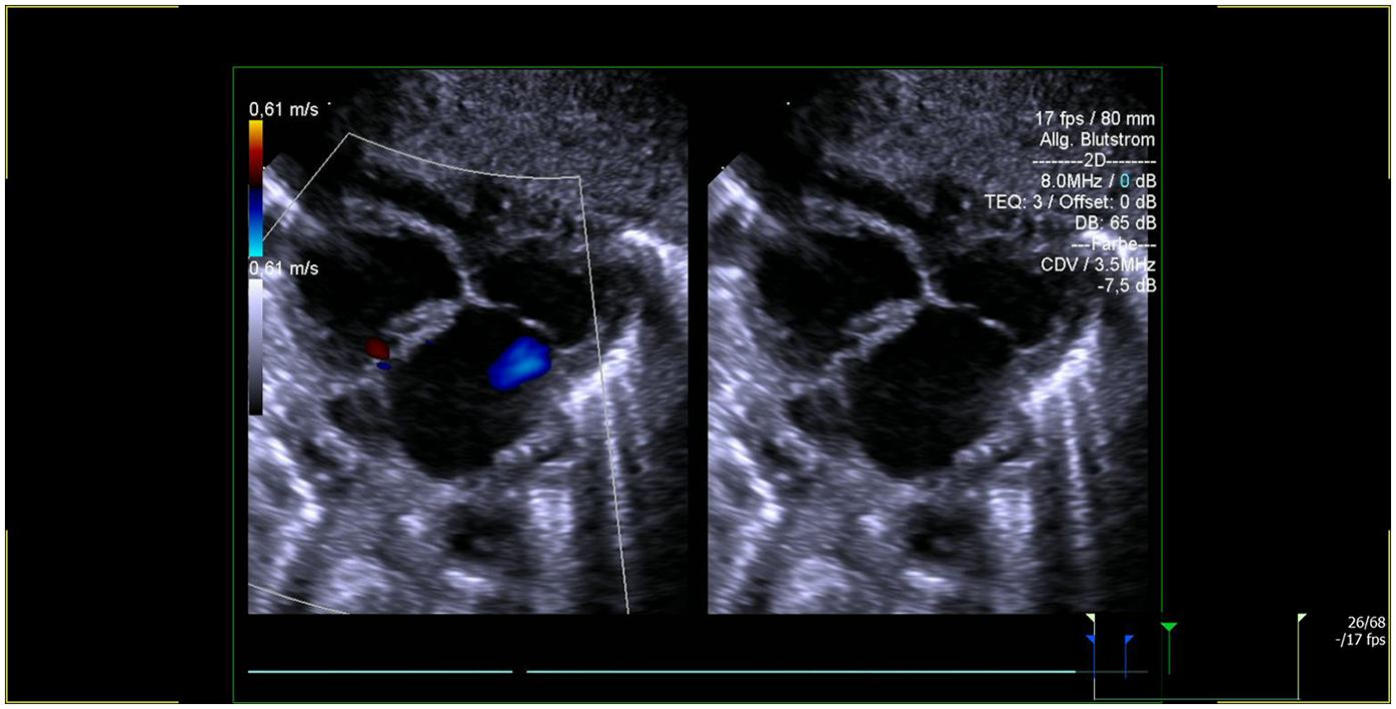
Transthoracic echocardiography, subcostal four-chamber view, of a thickened interatrial septum with a restrictive FO.

**Figure 5:**
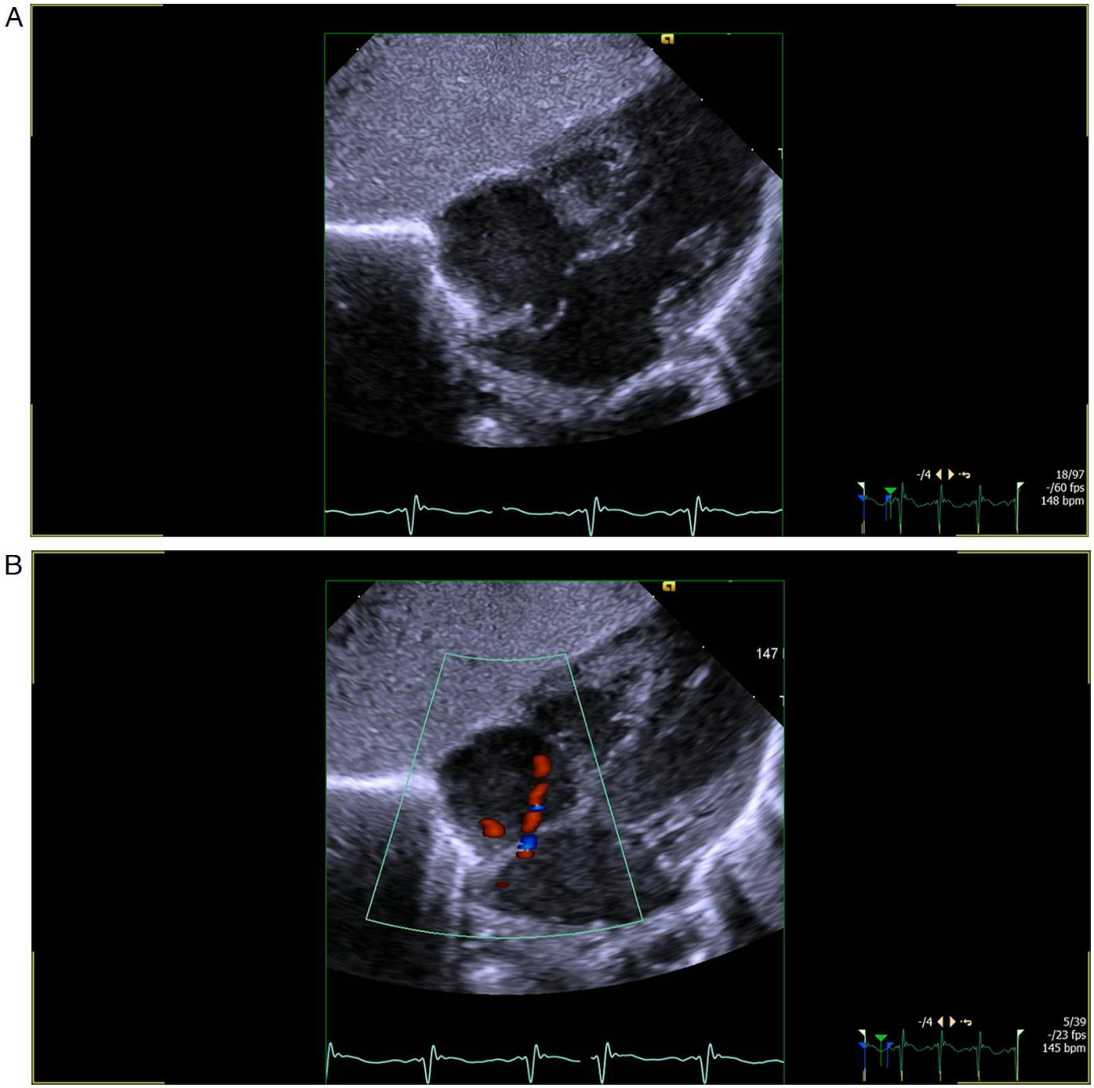
**(A, B)** Transthoracic echocardiography, subcostal four-chamber view, of a thin interatrial septum with a restrictive FO.

In this series, echocardiography demonstrated FO closure in 6 out of 10 patients. However, autopsy revealed a small ectopic atrial septal opening in a deceased patient in whom we were not able to demonstrate a shunt across the interatrial septum (Figure 1). In five of these six patients, a thickened interatrial septum was noted (Figures 2, 3). Nevertheless, a complete closure was also diagnosed in a patient with a thin interatrial septum. FO restriction was demonstrated using color flow Doppler echocardiography in four patients, with two having a thick interatrial septum and two a thin interatrial septum (Figures 4, 5).

**Figure 6:**
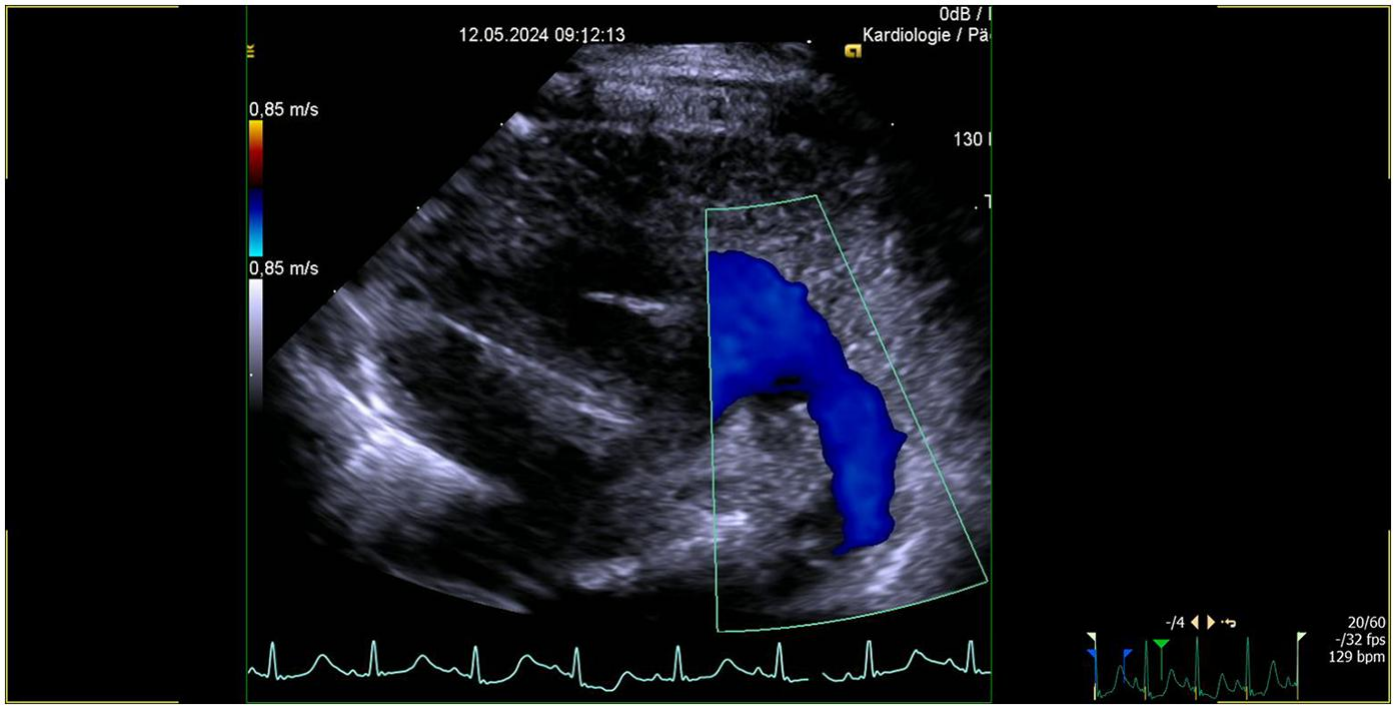
Transthoracic echocardiography, parasternal ductal view, of a predominantly right-to-left ductal shunt.

**Figure 7:**
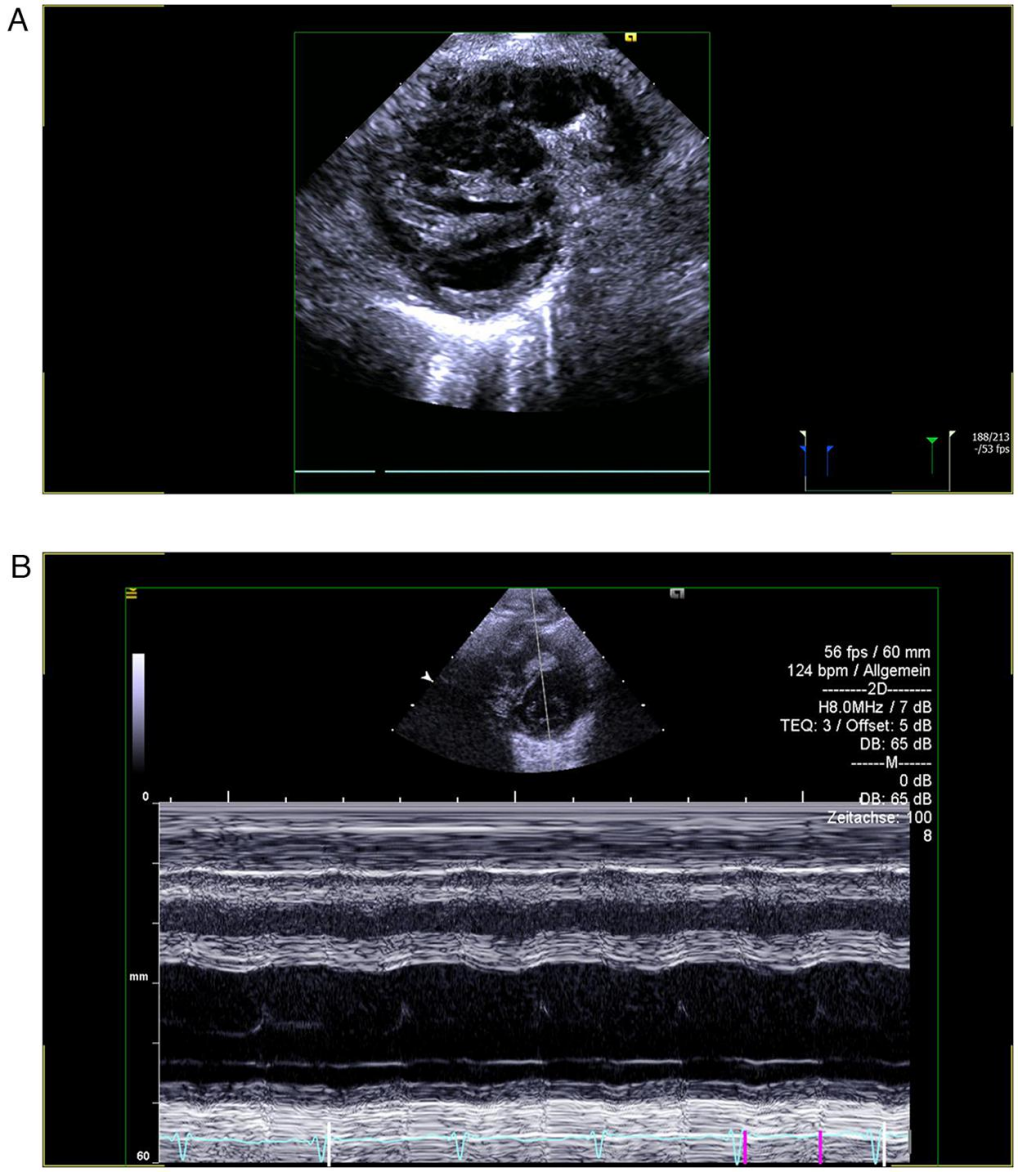
**(A, B)** Transthoracic echocardiography, two-dimensional and M-mode, of normal right ventricular contractility, flattening of the interventricular septum, and virtual akinesia of the left ventricular free wall.

**Figure 8:**
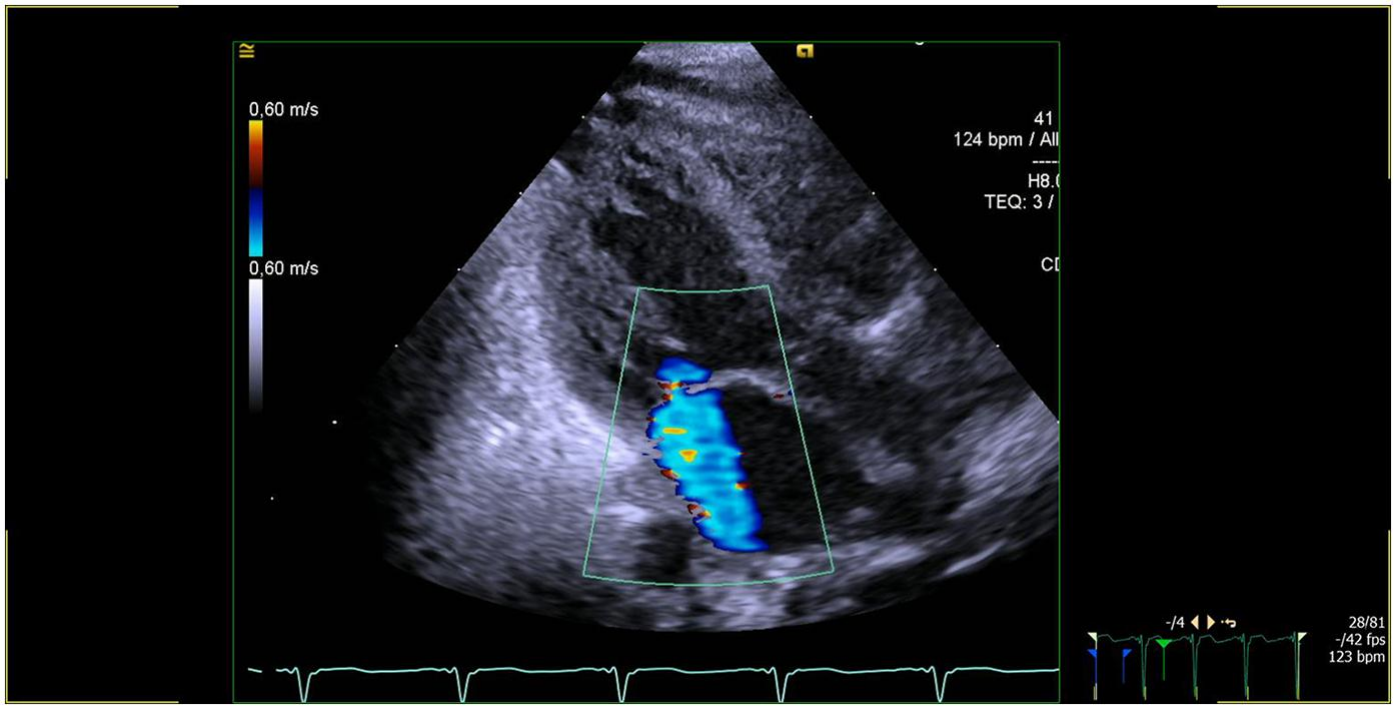
Transthoracic echocardiography, apical long axis view, of normal right ventricular contractility and severely impaired left ventricular contractility associated with moderate mitral regurgitation.

In all of our patients, the characteristic echocardiographic pattern at presentation consisted of PPHN and markedly reduced LV contractility (Figures 6–8). In these neonates, PPHN has already been reported (1, 2). In fetuses with FO closure or restriction, the right-to-left FO shunt is either completely obstructed or very diminished, leading to a substantial increase in RV output. Despite the right-to-left DA shunt, fetal pulmonary blood flow is increased, leading to remodeling of pulmonary circulation. This process plays a critical role in the development of PPHN and therefore, PPHN was an expected finding in our cohort. In contrast, associated LV dysfunction has been less well-characterized thus far.

Uzun et al. observed impaired function of both ventricles in two neonates and impaired LV function in 1 of 23 neonates (1). Kadish et al. also noted impaired LV and RV function in 1 of 34 patients, respectively (7). In contrast, eight of our patients exhibited severely impaired LV contractility, while in the remaining two patients, LV contractility was moderately impaired. Conversely, RV function was preserved in all our patients. Virtual akinesia of the LV free wall and interventricular septum flattening precluded precise quantification of systolic and diastolic ventricular function. We assume that LV filling (preload) is not sufficient to sustain normal LV ventricular contractions in these patients. Thus, it appears that reduced pulmonary venous return due to PPHN, together with FO closure or restriction, precludes adequate LV filling. In contrast, the preserved RV function in all of our patients probably eliminates myocardial ischemia as an explanation for LV impairment.

At NICU admission, LV output was low in this group of patients, reflected in increased lactate levels in all of them. In the most severely affected patients, the antegrade flow through the aortic valve was initially barely detectable by color flow Doppler echocardiography. Thus, in addition to the predominantly right-to-left DA shunt, a retrograde flow in the aortic arch was demonstrated in the most severely affected neonates. In order to maintain DA-dependent systemic perfusion, prostaglandin infusions were administered and were the mainstay of our initial treatment strategy. Simultaneously, inotropic and PAH therapy were initiated. We observed a rapid improvement of the LV contractility and accompanying mitral regurgitation with a complete normalization within 4 days of treatment, further supporting our assumption of functional LV myocardial impairment. Thereafter, we gradually decreased and stopped the inotropic therapy and intensified the PAH therapy. A predominantly left-to-right DA shunt was noted up to 8 days after the initiation of treatment, prompting us to discontinue the prostaglandin infusions. Subsequently, we continued only with PAH therapy.

Nine of the 10 patients in this series survived, despite severe clinical presentations in the majority of them. Furthermore, neurological outcomes, without clinical or neuroimaging evidence of neurological sequelae, were good in seven of the nine surviving patients. However, a viable option in neonates with an extremely difficult presentation is veno-arterial extracorporeal membrane oxygenation and this should be taken into consideration in these patients.

## Conclusion

Isolated prenatal FO closure or restriction may lead to severe clinical presentation after birth, marked by central or differential cyanosis and respiratory distress. Echocardiography typically reveals PPHN together with severely reduced LV contractility. This unique combination of findings should prompt a precise evaluation of the interatrial septum. A timely diagnosis of this reversible condition can facilitate management and may improve outcomes. This distinctive presentation and ensuing clinical course have not been reported thus far and suggest a hitherto under-recognized clinical condition.

## Data Availability

The original contributions presented in the study are included in the article/Supplementary Material, further inquiries can be directed to the corresponding author.
